# The Need for Full Integration of Snakebite Envenoming within a Global Strategy to Combat the Neglected Tropical Diseases: The Way Forward

**DOI:** 10.1371/journal.pntd.0002162

**Published:** 2013-06-13

**Authors:** José María Gutiérrez, David A. Warrell, David J. Williams, Simon Jensen, Nicholas Brown, Juan J. Calvete, Robert A. Harrison, for the Global Snakebite Initiative

**Affiliations:** 1 Instituto Clodomiro Picado, Facultad de Microbiología, Universidad de Costa Rica, San José, Costa Rica; 2 Nuffield Department of Clinical Medicine, John Radcliffe Hospital, University of Oxford, Oxford, United Kingdom; 3 School of Medicine & Health Sciences, University of Papua New Guinea, Port Moresby, Papua New Guinea; 4 Australian Venom Research Unit, Department of Pharmacology, University of Melbourne, Parkville, Victoria, Australia; 5 Royal Brisbane and Women's Hospital, Herston, Queensland, Australia; 6 Instituto de Biomedicina de Valencia, Consejo Superior de Investigaciones Científicas (CSIC), Valencia, Spain; 7 Alistair Reid Venom Research Unit, Liverpool School of Tropical Medicine, Liverpool, United Kingdom; Faculty of Medicine, University of Kelaniya, Sri Lanka

Snakebite envenoming constitutes a serious medical condition that primarily affects residents of rural communities in Africa, Asia, Latin America, and New Guinea [1], [2]. It is an occupational, environmental, and domestic health hazard that exacerbates the already impoverished state of these communities [3]. Conservative estimates indicate that, worldwide, more than 5 million people suffer snakebite every year, leading to 25,000–125,000 deaths, while an estimated 400,000 people are left with permanent disabilities [4]–[7]. Eight thousand amputations are thought to be performed annually in Africa alone [8]. However, community-based surveys illustrate that the actual burden of human suffering is likely to be even greater [9], [10]. Despite this global impact, snakebite has received little attention from the global health community, the pharmaceutical industry, governments, and public health advocacy groups, and has a disappointingly low priority in the global health research agenda. As a consequence, the paucity of health programs addressing snakebite at national, regional, and global levels allows deaths or maimings of snakebite victims to continue. This burden of suffering could be significantly reduced because effective preventive and therapeutic resources are available, but, because of systemic neglect, they are not delivered in many regions. There has been progress in highlighting the neglect of snakebite. Thus, the inclusion of snakebite in the WHO list of Neglected Tropical Diseases (NTDs), and the development of initiatives by the WHO and its regional offices [1], [11] as well as by the Global Snakebite Initiative (GSI) [6] and other efforts at national and regional levels, have improved the global awareness of this disease. However, the impact of these projects has been rather limited, particularly in light of the progress made in control of the helminthic NTDs.

Global efforts launched in the last decade to confront NTDs have recruited the important support of the World Health Organization (WHO), governments, diverse funding agencies, and other advocacy groups/foundations [12], [13]. As a result, there is a growing awareness of the sociomedical importance of this group of ancient human scourges. Several strategies are being implemented to reduce the burden of these diseases [13] within the framework of the Millennium Development Goals (MDGs). A significant achievement has been the conceptualization of NTDs as a group of health problems that share many common demographic, sociological, epidemiological, and clinical features. Implementing integrated initiatives conducted by advocates, involving research and development, control, treatment, and attention to the needs of affected populations, is now a primary strategy to reduce disease burden. Regrettably, snakebite, despite being included in the 2009 WHO list of NTDs, has not been incorporated into these globally coordinated efforts to reduce the impact of the NTDs. The reasons for this omission are diverse and are probably based upon the perception that, because snakebite is not an infectious disease, the strategies for its alleviation do not fit within the strategies used to combat the “typical” NTDs. This perception is misleading, since snakebite epitomizes the main features that characterize NTDs [13]:

1. Snakebite causes significant rates of mortality, morbidity, disfigurement [6], and chronic psychological sequelae [14], and incurs a heavy loss of productivity due to physical disability. Since impoverished rural farmers are the group at highest risk [3], snakebite exerts a direct economic and social impact on families and communities and thereby significantly contributes to the prevailing vicious cycle of poverty and inequity.
2. Since snakebite mainly afflicts low-profile, rural populations that lack a political voice, victims cannot influence regional and national administrative and political policy makers, and their needs remain largely unheard and politically neglected.
3. Snakebite does not represent a health risk to high-income peoples and countries. This contributes to the negligible interest shown by governments to combat this problem with the financial and political resources appropriate to that task.
4. Snakebite causes stigma and discrimination, especially in people suffering from venom-induced permanent physical deformity and disability, as well as from amputations and other surgical procedures employed in the management of these complications [8]. This affects working performance and greatly limits the chances of victims' finding jobs and leading productive and fulfilling lives. In addition, since a high proportion of cases occur in children, snakebite may have profound implications for their development, education, and future opportunities, blighting their entire lives.
5. The true rates of snakebite-induced morbidity and mortality are still largely unknown in many regions of the world because estimates are based mostly on extrapolations of hospital statistics. Recent national community-based surveys have highlighted the fact that the actual magnitude of this disease is much greater than was previously thought because many snakebite victims never manage to reach hospitals and therefore remain unrecorded and invisible to the health system [9], [10]. The current estimate of 20,000 to 94,000 deaths caused by snakebite annually [5] is therefore bound to be an underestimate, making the burden of mortality from snakebite much higher than for other NTDs [6].
6. Snakebite has been largely omitted from research agendas and does not feature as a listed research priority for any health funding agency. Despite significant advances in the biochemical and toxicological understanding of snake venoms, including the realization of their potential as pharmacological agents, there are serious deficiencies in our knowledge of the epidemiological and clinical features of snakebite envenomings in many countries. There has also been negligible funding for research to improve the technologies for antivenom manufacture—the only validated therapy for snakebite envenoming. Likewise, topics related to economic impacts, public health policies, and cultural perceptions of snakebite have failed to attract the attention of research groups and their funders.
7. The tragedy of snakebite is that effective solutions already exist but are not being delivered in many countries. Timely administration by a trained practitioner of effective and appropriate antivenoms can be expected to prevent most deaths and sequelae resulting from these envenomings. Although approved methods for antivenom production are readily available in the public domain, antivenoms are neither available nor accessible in many regions of the world [15].

Some distinctions between snakebite and the other NTDs pose significant challenges to establishing effective snakebite control programs. Thus, because it is not an infectious disease, there is no potential for elimination or eradication of snakebites, unlike the expectation for most other NTDs. Highly effective and logistically efficient mass vaccination or administration of antihelminthics, antibiotics, and other interventions, such as vector control, and provision of safe food, water, and sanitation [13], are not applicable to snakebite envenoming. Furthermore, unlike the near global effectiveness of most antihelminthics and antibiotics, snakebite envenoming therapy is regionally specific and this limits the implementation of “economics of scale” to antivenom production.

Nevertheless, the fact that snakebite envenoming coexists with bacterial, viral, and parasitic NTDs in many rural settings suggests patterns of comorbidity that are amenable to integrated intervention. There are several features of snakebite control that are similar to the principal aspects used to combat the infectious NTDs, indicating that incorporation of snakebite prevention, treatment, and rehabilitation resources into the strategies to fight NTDs would offer a great health benefit to vulnerable communities. For example, encouraging the wearing of appropriate shoes, the use of a torch after dark, sleeping under an insecticide-impregnated bed net, and speeding patient transport to medical care in remote areas using trained volunteer motorcyclists (S.K. Sharma, personal communication, 2011) are all effective in reducing the incidence of snakebite [16]. These approaches would also reduce the burden of soil-transmitted helminthic infections, tropical and Buruli ulcer, podoconiosis, malaria, and kala-azar and other vector-borne infections. Most of the key elements of the WHO Global Plan to Combat Neglected Tropical Diseases 2008–2015 [13] also apply to reduce the burden of snakebite envenoming.

## The Way Forward: Toward a Globally Integrated Strategy

The global struggle to reduce the impact of snakebite envenoming should be based on an integrated approach encompassing diverse interventions. The key actions to achieve this should be coordinated with the more general efforts to combat NTDs, such as:

1. Improving health information systems to generate reliable disease-burden data in regions of high NTD incidence. These data would allow the design of knowledge-based strategies for effective prevention and treatment of all the NTDs [17], including snakebite.
2. International research efforts dedicated to achieving a better understanding of the biological epidemiology of the NTDs would also serve to improve understanding of the systematics and venom composition of the snake species of greatest medical importance, thereby fostering the development of antivenoms with broader coverage of snake species and geographical areas [18].
3. Improving the availability and accessibility of safe and effective NTD treatments. This difficult task is being very effectively addressed for the helminthic NTDs but remains a formidable challenge for many others. In the case of snakebite treatment, it involves the strengthening of current manufacturing laboratories, the commitment of large laboratories to generate more effective antivenoms, and the promotion of technology transfer projects aimed at enabling countries with a high burden of snakebites to produce their own antivenoms. There is a self-perpetuating cycle that has resulted in a decline in the production of antivenoms, especially for sub-Saharan Africa. Inadequate financial support for antivenom production and the poor quality of some products has caused loss of confidence, a reduced market, a consequent drop in production, and increased prices, which in turn contribute to further reduction in availability and accessibility, and an alarming increase in the unscrupulous marketing of inappropriate products [19]–[21]. The net result has been a serious crisis in the access to antivenoms in various regions of the world. The solution to this problem has to be based on: (a) the generation of increased volumes of safe and effective antivenoms, (b) the improvement of the capacity of national regulatory agencies to ensure the safety and efficacy of antivenoms, (c) the allocation of national and international resources for the regular purchase of antivenoms, and (d) the design of marketing and purchasing strategies that guarantee access to affordable antivenoms for the health systems of developing countries. This resolution of the snakebite problem should go hand in hand with the strengthening of public health systems, especially with the provision of health services to vulnerable populations in areas of high coincidence of all the NTDs.
4. Ensuring that the health workforce in areas of snakebites is appropriately trained in the clinical management of these diseases. Establishing and maintaining “best clinical practice” education programs for all the NTDs in high-risk regions would improve health outcomes significantly. In many cases, these education packages are already available but inadequately distributed. Thus, the WHO has provided regional snakebite-management guidelines [22], [23] and relevant teaching materials for nurses and dispensers as well as medical officers. These activities can incorporate information and communication technologies of various sorts in a creative manner to maximize effectiveness.
5. Attention and follow-up for people suffering from physical and psychological sequelae secondary to NTDs. Snakebite, like some of the other NTDs, causes substantial disfigurement and disability that frequently results in chronic psychological morbidity [13], [24]. Given the comorbidity and geographical coexistence of these NTDs, there is an urgent and compelling need for an integrated strategy, focusing on wound healing and psychological support, to alleviate these physical and psychological complications.
6. Implementation of preventive and educational campaigns to reduce the incidence of NTDs and to promote effective first aid intervention. Again, these campaigns would benefit victims of all the NTDs, including snakebite victims. The involvement of local communities in the design and performance of these activities is of paramount relevance to increasing the likelihood of community compliance.

The fulfillment of these tasks demands an integrated, interprogrammatic, and intersectorial strategy at the global level, involving a wide spectrum of active protagonists, such as: (a) the medical and scientific community, (b) technology development groups, (c) antivenom manufacturers, (d) national and international public health authorities, (e) advocacy groups, international partners, and nongovernmental organizations working in the public health sector, and (f) diverse community organizations and local initiatives in the regions where snakebites are frequent [15]. The implementation of this ambitious strategy, which is being promoted by the GSI (www.snakebiteinitiative.org) and other institutions and organizations, should be integrated with other programs designed to combat NTDs. To quote from the Director-General of the WHO's address (2007) to the Regional Committee for Africa: “Last year, WHO launched an integrated strategy for the management of several of the neglected tropical diseases, all of which disproportionately affect the poorest of the poor in Africa. Instead of a host of individual programs going their separate ways, we now have a unified strategy that simplifies drug distribution, reduces duplication, and lessens some of the demands on health systems and staff.” The incorporation of the proposed snakebite initiatives within the general struggle against all the NTDs will result in a significant and more logistically efficient reduction of human suffering.
